# Development and Characterization of 1,906 EST-SSR Markers from Unigenes in Jute (*Corchorus* spp.)

**DOI:** 10.1371/journal.pone.0140861

**Published:** 2015-10-29

**Authors:** Liwu Zhang, Yanru Li, Aifen Tao, Pingping Fang, Jianmin Qi

**Affiliations:** Key Laboratory of Ministry of Education for Genetics, Breeding and Multiple Utilization of Crops, College of Crop Science, Fujian Agriculture and Forestry University, Fuzhou, China; NBPGR, INDIA

## Abstract

Jute, comprising white and dark jute, is the second important natural fiber crop after cotton worldwide. However, the lack of expressed sequence tag-derived simple sequence repeat (EST-SSR) markers has resulted in a large gap in the improvement of jute. Previously, de novo 48,914 unigenes from white jute were assembled. In this study, 1,906 EST-SSRs were identified from these assembled uingenes. Among these markers, di-, tri- and tetra-nucleotide repeat types were the abundant types (12.0%, 56.9% and 21.6% respectively). The AG-rich or GA-rich nucleotide repeats were the predominant. Subsequently, a sample of 116 SSRs, located in genes encoding transcription factors and cellulose synthases, were selected to survey polymorphisms among12 diverse jute accessions. Of these, 83.6% successfully amplified at least one fragment and detected polymorphism among the 12diverse genotypes, indicating that the newly developed SSRs are of good quality. Furthermore, the genetic similarity coefficients of all the 12 accessions were evaluated using 97 polymorphic SSRs. The cluster analysis divided the jute accessions into two main groups with genetic similarity coefficient of 0.61. These EST-SSR markers not only enrich molecular markers of jute genome, but also facilitate genetic and genomic researches in jute.

## Introduction

Jute (*Corchorus* spp.) is a diploid plant of the Tiliaceae family (2n = 14) and has about 60 species under the genus, out of which white jute (*C*. *capsularis*) and dark jute (*C*. *olitorius*) are two commercially cultivated species [1–3]. It is the second important crop for natural fiber production after cotton in the word. Jute mainly cultivated in India, China, Bangladesh, Malaysia, South Africa, Thailand, United States and southeast Europe [1]. Jute fibers, stripped from the stem, are mainly used for rope, coarse cloth and paper. In addition, jute can produce many valuable by-products such as clothing-grade cloth, insulation, engineered wood, packing material, animal feed, seed oil and potential biofuel [4–6]. Therefore, jute has received much attention in China, India, Malaysia, the United States, Mexico, Italy and so on.

Simple sequence repeat (SSR) markers or microsatellite markers have been widely used in gene mapping, genetic diversity assessing, marker-assisted breeding (MAB) and so on [2–6]. To obtain the SSR information, some SSRs have been designed based on genomic sequences of jute. According to Mir et al. [7], four independent SSR-enriched genomic libraries were constructed and sequenced to develop 2,469 SSRs in dark jute. Moreover, the same group reported an additional set of 607 novel SSR based on the same genomic libraries [8]. However, few studies on SSR development had been conducted in white jute although there are some researches on SSR development in dark jute [7–8].

From the resource of sequences used for SSR identification, SSR can be divided into genomic SSRs (originated from random genomic sequence) and expressed sequence tags (ESTs) SSR. The SSR developed by Mir et al. [7] belongs to genomic SSRs. As is known, EST-SSRs are usually located in functional genes which linked with certain important traits, EST-SSRs are more useful than genomic SSRs in the improvement of jute. Thus, it is imperative to develop new large-scale EST-SSRs in jute.

Compared with large-scale sequencing of genome or cDNA libraries, next-generation sequencing (NGS) is a relatively less expensive and laborious way to obtain sequences for SSRs identification. To obtain the SSR information, abundant transcriptome sequences based on NGS have been generated from natural fiber crops [9–10]. Liu et al. [9] developed 1,827 SSRs using transcriptomic sequencing in ramie. Gao et al. [10] identified 4,577 EST-SSR from 3624 ESTs in flax. There is an urgent need to develop EST-SSR in jute using NGS.

Unigenes (unique genes), which refer to a non-redundant set of gene-oriented clusters of ESTs and other mRNA sequences, provide a valuable and cost-effective source for the development of SSR markers. Previously, Huangma 179, a control variety of white jute from the official variety registry in China, was used to conduct the transcriptome sequencing and the sequences of 48,914 unigenes were deposited in GenBank. In this study, the aim was to develop and characterize EST-SSRs from these 48,914 unigenes. The newly developed SSRs would not only enrich molecular markers, but also facilitate genetic researches in jute.

## Materials and Methods

### Plant Materials and DNA Extraction

The 12 jute accessions,which were provided by Genetics and Natural Fiber Breeding Laboratory of Fujian Agriculture and Forestry University, had different genetic backgrounds and were used as tested materials (Table 1). Among them, six accessions (Huangma 179, Aidianyehuangma, Qiongyueqing, D-154, and Yueyuan 5) were white jute and six accessions (JRC-212, BL/106CG, Yunye 1–1, Kuanyechangguo, Tianma, Maliyeshengchangguo, and JRC/551) were dark jute. Two accessions (Huangma 179 and Kuanyechangguo) are two control varieties from the official variety registry in China, out of which Huangma179 was used to conduct transcriptome sequence of bast fiber.

**Table 1:** Names and origins of 12 tested germplasms in jute.

Code	Name	Origin	Type	Species
1	Huangma179	Fujian, China	Cultivated	*C*. *capsularis*
2	Aidianyehuangma	Henan, China	Wild	*C*. *capsularis*
3	Qiongyueqing	Hunan, China	Cultivated	*C*. *capsularis*
4	D-154	India	Cultivated	*C*. *capsularis*
5	Yueyuan 5	Guangdong, China	Cultivated	*C*. *capsularis*
6	JRC-212	India	Cultivated	*C*. *capsularis*
7	BL/106CG	Kenya	Cultivated	*C*. *olitorius*
8	Yunye 1–1	Yunnan, China	Wild	*C*. *olitorius*
9	Kuanyechangguo	Hunan, China	Cultivated	*C*. *olitorius*
10	Tianma	Yunnan, China	Relative wild	*C*. *olitorius*
11	Maliyeshengchangguo	Mali	Wild	*C*. *olitorius*
12	JRC/551	Nepal	Cultivated	*C*. *olitorius*

All the 12 jute accessions were planted on May 1st, 2014 in the experimental farm of Fujian Agriculture and Forestry University, Fuzhou, China. Genomic DNA from the 12 accessions was extracted from 30-day-old seedlings using a modified cetyltrimethyl ammonium bromide (CTAB) method [11]. The details about the modification of this CTAB method are as follows: (1) Add one volume of chloroform:isoamyl alcohol (24:1) to the sample, shake by hand thoroughly for approximately 20 seconds; (2) Centrifuge at room temperature for 10 minutes at 12,000 × g. Carefully remove the upper aqueous phase, and transfer the layer to a fresh tube. The DNA was diluted to the concentration of 50 ng/*μ*L with double distilled H_2_O before polymerase chain reaction (PCR).

### Source of Sequences and SSR Identification

A total of 48,914 jute unigene sequences were derived from transcriptomic sequence at a vigorous stage in an elite white jute cultivar Huangma 179 and deposited at the NCBI Sequence Read Archive (SRA, http://www.ncbi.nlm.nih.gov/Traces/sra) under the accession number SRP060467 vide BioSamples SRS980707. Using software Primer 3.0 [12], 1,906 pairs of primers (designated as CcES) (S1 Table) were designed based on the SSR sites screened by the online program SSRPrimer (http://hornbill.cspp.latrobe.edu.au/ssrdiscovery.html). The criteria for SSR selection were set at six repeats for di-nucleotides, four repeats for tri-nucleotides, three repeats for tetra- and penta- nucleotides. Among 1,906 CcES, 113 and 3 SSRs located in genes that encode transcription factors (TFs) and celllulose synthases (CesAs) respectively, were selected for subsequent analysis (S2 and S3 Tables). The TF families involved in SSRs were WRKY, MYB-related, bHLH, AP2-EREBP, AUX/IAA, GRAS, MYB, SBP and so on. The 3 CesAs involved in SSRs were CesA1, CesA6 and CesA10 respectively. All these 116 primers were synthesized by Shanghai Biological Technology Co., Ltd.

### Functional Annotation of Unigenes Contained SSR Loci

Gene function was annotated using BLASTX (Basic Local Alignment Search Tool for sequences translated in three frames) with an E-value threshold of 10^−5^ in the non-redundant protein database (non-redundant protein sequences, http://www.ncbi.nlm.nih.gov).

### PCR Reaction and Amplification for SSR

To test their polymorphism, a sample of 116 pairs of SSRs, out of which 113 and 3 SSRs located in genes that encode TFs and CesAs respectively, were amplified in 12 jute accessions. The criteria for SSR polymorphism were polymorphic between at least two accessions. PCR amplifications were performed in a volume of 10μl containing 50 ng·μl^−1^ DNA 2.0 μl, 10 μmol·μl^−1^ left primer 0.5 μl, 10 μmol·μl^−1^ right primer 0.5 μl, 0.5 U·μl^−1^ *Taq* polymerase 0.1 μl, 10 mmol·l^−1^ dNTPs 0.2 μl, 10×PCR buffer 1 μl, 50 mmol·l^−1^ Mg^2+^ 0.8μL, and dd H_2_O 4.9 μl. The PCR procedure, electrophoresis and silver staining were as described by Zhang *et al*.[13].

### Cluster Analysis

The polymorphic bands from SSRs were coded as the presence (1) or absence (0) of bands. Jaccard's genetic similarity coefficient (GSC) comparing all pairs of the 12 jute accessions was calculated on the basis of unweighted pair group method of arithmetic means (UPGMA), and a dendrogram was constructed by NTsys V2.02 [14]. Estimation of polymorphism information content (PIC), which is a measure of expected heterozygosity, was estimated by PowerMarker 3.51 [15].

## Results

### Development of EST-SSRs from Unigenes

A total of 48,914 jute unigenes' sequences derived from the transcription sequence, representing a total length of 44.35Mb, were analyzed for SSR-containing sequences. 1,906 SSRs, designated as CcES markers hereafter, with repeat lengths of at least12 nucleotides were identified in 1,410 of the 48,914 unigenes (Table 2, S1 Table). The functions of these unigenes that contained SSRs were annotated using BLASTX (E-value < 10^−5^) queries in the database of nr (non-redundant protein sequences) (S2 Table). However, 295 (20.9%) of these unigenes had no annotation of gene so far. Among the 1410 unigenes examined, 329 sequences contained more than 1 SSR. This means that 2.88% of 48,914 unigenes contained at least one SSR. The 1906 SSRs included 229 di-, 1,085 tri-, 411 tetra-, and 181 penta-nucleotide repeats.

**Table 2:** Frequencies of EST-SSR number based on unigenes in jute.

Items	Number
Total number of sequences examined	48,914
Total number of unigenes examined	1,410
Total size of examined sequences (kb)	44,354
Total number of identified SSRs	1906
Dinucleotide	229 (12.0%)
Trinucleotide	1085 (56.9%)
Tetranucleotide	411 (21.6%)
Pentanucleotide	181 (9.5%)
Number of sequences containing more than 1 SSR	329

### Frequency and Length Variation of EST-SSRs from Unigenes

Among 1906 SSRs, the di-, tri-, tetra-, and penta-nucleotide repeat types have frequencies of 12.0%, 56.9%, 21.6%, and 9.5% respectively (Table 2). Of these markers, the tri-nucleotide repeat types are the highest abundant repeat types and the di- and tetra-nucleotide repeat types are higher abundant ones. The penta-nucleotide repeat types have the lowest frequency. In the average length of different repeat types, the longest average length was 17.9 bp (penta-nucleotide), followed by 15.5 bp (di-nucleotide) and15.2 bp (tri-nucleotide). The tetra-nucleotide repeat types have the shortest average length (13.9 bp) (Table 3).

**Table 3:** Frequencies of 1,906 EST-SSR repeat types based on unigenes in jute.

Repeat type	Repeat type	Numbers	Total length(bp)	Average length(bp)
Dinucleotide		229 (12.0%)	3555	15.5
	AG/CT	79	1178	14.9
	AT/AT	26	417	16.0
	TC/GA	69	1072	15.5
	TA/TA	34	525	15.4
	TG/CA	11	217	19.7
	others	10	146	14.4
Trinucleotide		1085 (56.9%)	16528	15.2
	AAC/GTT	25	369	14.8
	AAG/CTT	80	1215	15.2
	AAT/ATT	36	555	15.4
	ACC/GGT	24	358	14.9
	AGA/TCT	103	1532	14.9
	AGC/GCT	41	596	14.5
	AGG/CCT	26	418	16.1
	ATA/TAT	40	563	14.1
	ATC/GAT	49	740	15.1
	ATG/CAT	44	680	15.5
	CAG/CTG	42	627	14.9
	CCG/CGG	22	316	14.4
	GAG/CTC	29	430	14.8
	GCC/GGC	30	446	14.9
	TAA/TTA	23	347	14.5
	TCA/TGA	65	984	15.1
	TCC/GGA	36	551	15.3
	TGC/GCA	39	552	14.2
	TGG/CCA	40	707	17.7
	TTC/GAA	147	2318	15.8
	TTG/CAA	43	686	16
	Others	101	1538	15.5
Tetranucleotide		411 (21.6%)	5702	13.9
	AAAG/CTTT	50	716	14.3
	AAAT/ATTT	17	243	14.3
	AAGA/TCTT	22	325	14.8
	AGAA/TTCT	32	468	14.6
	ATAA/TTAT	10	148	14.8
	ATTA/TAAT	14	201	14.4
	TTTA/TAAA	18	240	13.3
	TTTC/GAAA	34	488	14.4
	TTTG/CAAA	14	173	12.4
	Others	200	2700	14.1
Pentanucleotide		181 (9.5%)	3238	17.9

In di-nucleotide repeats, the highest abundant repeat types were (AG/CT)n and (TC/GA)n. Intri-nucleotide repeats, (TTC/GAA)n, (AGA/TCT)n and (AAG/CTT)n types were the most common, followed by (TCA/TGA)n. In tetra-nucleotide repeats, the most abundant repeat types were (AAAG/CTTT)n, (TTTC/GAAA)n and (AGAA/TTCT)n. From the frequency of repeat types, it could be concluded that the SSRs, which contained AG-rich or GA-rich nucleotide repeats, are considered as the predominant types in jute (Table 3).

### Detection of Polymorphism

Because functional markers are useful in the breeding program, it is valuable for jute to identify SSRs located in functional genes, i.e., transcription factors and cellulose synthase. By searching the annotation of 1,410 unigenes with the expression of transcription factor and cellulose synthase, 113 and 3 SSRs, located in genes that encode TFs and CesAs respectively, were identified (S2 and S3 Tables). To assess the quality of these EST-SSRs and evaluate their polymorphism, the 116 SSRs were amplified in 12 diverse jute accessions using PCR (S3 Table). The gel electrophoresis results showed that 97 pairs of primers (83.6%) were successfully amplified at least one fragment from the jute genome and were polymorphic between at least two accessions. The amplified bands of the primer CcES1743 are shown in S1 Fig as an example. The number of polymorphic bands produced by each primer varied from 1 to 4 and the average was 1.4. Of these successfully amplified SSRs, 28 SSRs (24.1%) amplified at least two polymorphic fragments and the PCR products were clear and stable fragments. The PIC varied from 0 to 0.5821 and the average was 0.0932.

### Cluster Analysis

**Fig 1:**
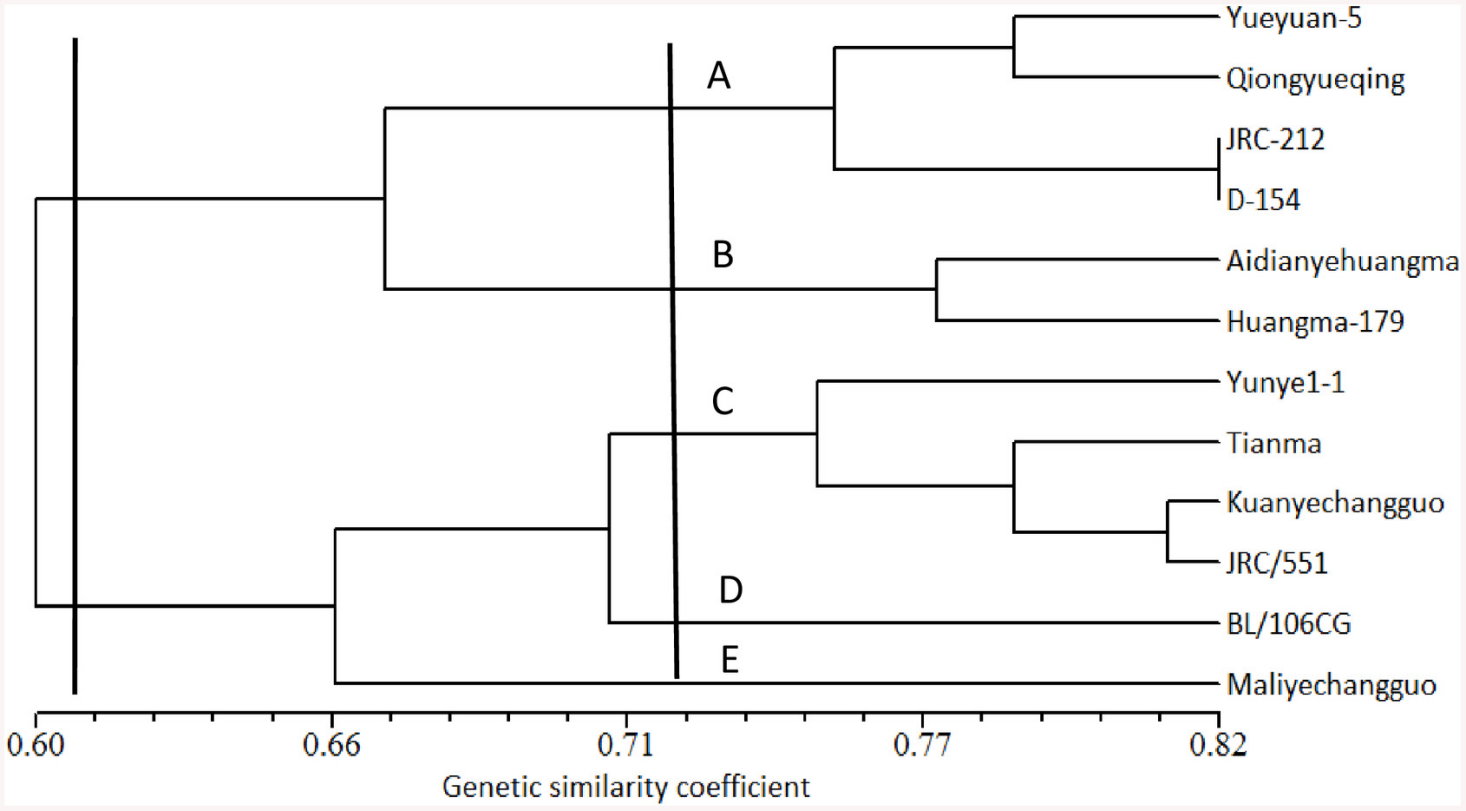
Dendrogram plot based on cluster analysis of 97 polymorphic EST-SSR markers by UPGMA. The 12 jute accessions classified into two main groups of dark and white jute, further into five subgroups.

To examine the genetic relationships among the 12 jute accessions, 97 SSRs, which showed polymorphism among at least two accessions, were collected (S3 Table). The total of polymorphic bands was134, with an average of 1.4 per primer. Out of the 12 jute accessions, the genetic similarity coefficient (GSC) ranged from 0.502 to 0.820, with the average of 0.653 (S4 Table; Fig 1). The lowest GSC (0.502) was observed between Huangma 179 (white jute) and JRC/551 (dark jute), followed by JRC-212 (white jute) and JRC/551 (dark jute). And the highest GSC (0.802) was found between JRC-212 and D-154, indicating that there is a close relationship between them. According to the pedigree analysis, D-154 is introduced from India in 1952 and JRC-212 is a pure line selected from D-154 in 1954.

Taking a GSC value of 0.61 as the threshold, the 12 jute accessions could be distinctly classified into two main groups: dark and white jute (Fig 1), indicating that there is relatively wide genetic variation between dark and white jute. When a GSC of 0.73 was used, the 12jute accessions were further classified into five subgroups (A, B, C, D and E). Out of subgroups, dark jute has two subgroups (A and B) while dark jute has three subgroups (C, D and E). The subgroups D contained 1 accession (BL/106CG) and the subgroups E contained 1 accession (Maliyeshengchangguo), which is worth mentioning because the lowest average GSC was observed. To enlarge the genetic variation in the cross-breeding program in jute, the useful parental lines, such as Huangma 179, JRC-212, JRC/551 and Maliyeshengchangguo, should be selected from different subgroups.

## Discussion

The SSR markers, which were applicable in QTL mapping and genetic studies, are still limited in jute so far [3,6]. Few EST-SSR markers derived from jute have been reported in addition to the development of EST-SSR from Genbank database in our group [13]. In a certain sense, the lack of SSR markers has resulted in a large gap in genetic studies. The main achievement in the present study is that 1906 EST-SSRs were identified from 48,914 unigenes' sequences, which greatly enriched the number of EST-SSRs in jute. To assess the quality of these EST-SSRs, a sample of 116 SSRs were collected and amplified in 12 diverse jute accessions. 97 pairs of primers were successfully amplified from the jute genome. Only 19 pairs of primers failed to amplify due to large introns present in the target amplicon or splice sites across in the designed primers [9, 16]. Nevertheless, the 83.6% successful amplification indicated that 1906 EST-SSRs developed in the present study are of good quality and could be amplified by PCR. As far as we know, it is the first successful development of EST-SSR markers on a large scale in jute, which will facilitate genetic mapping and comparative genetic studies.

From the frequency of the EST-SSR markers in jute, we could see that 3.90% of 48,914 unigenes contained at least one SSR. This frequency is lower than that in coffee (18.5%) [17] and wheat (7.41%) [18], but higher than that in barley (2.8%) [19]. This observation suggests that EST-SSRs are prevalent in jute. Among the 1906 EST-SSRs, the di-, tri-, tetra-, and penta-nucleotide repeat types have the frequencies of 12.0%, 56.9%, 21.6%, and 9.5% respectively. Tri-nucleotide repeat types were the abundant repeat types, which was in accordance with the results reported in other higher plants, such as rapeseed [20], ramie [9] and so on. In di-nucleotide repeats, the abundant repeattypes were (AG/CT)n and (TC/GA)n. Intri-nucleotide repeats, (TTC/GAA)n, (AGA/TCT)n and (AAG/CTT)n types were the most common, followed by (TCA/TGA)n. This trend suggests that AG-rich or GA-rich nucleotide repeats are considered as the predominant types in jute. According to Mir et al. [7], four independent SSR-enriched genomic libraries, i.e. (AC/GT)n,(AG/CT)n, (AAC/GTT)n and (AAG/CTT)n, were constructed to develop SSR. From the point of the repeat types, AG-rich nucleotide repeats were involved in this previous study [7]. Taken together, 1906 EST-SSRs in the present study, which contained AG-rich and GA-rich nucleotide repeats, is a salutary complement to develop SSR in jute.

From the resource of sequences used for SSR identification, the genome sequence contains introns and exons while EST contains exons. Since introns were seldom subjected to the process of selection compared with exons, introns retained more polymorphic sequences than exons. Thus, EST-SSRs tended to have lower PIC values than genomic SSR. As reported by our previous studies [13], the average PIC of genomic SSR is higher than that of EST-SSR. The low average PIC (0.0932) for these 116 selected primers in this study were in accordance with this finding. Among different molecular markers, SSRs are considered as a desirable marker for MAB, because SSRs have the advantages of Mendelian codominant inheritance, rapid and convenient detection. Zhang et al. [21] used three SSRs linked to a fiber strength QTL (*QTLFS1*) to increase the fiber strength of commercial cultivars by MAB. When genes are identified to regulate important agronomical traits using forward and reverse genetic technologies, the SSRs in these genes can be regarded as functional markers in the breeding program [22]. TFs regulate a lot of biological processes, i.e., responses to environmental stimuli, maintenance of metabolic and physiological pathways [23], which meant that TFs play an important role in regulating gene functions at the mRNA level. In this study, some TF families contained SSR loci are secondary wall-associated MYB TFs. As is known, MYB TFs are master switches regulating a cascade of downstream transcription factors and lead to activation of the secondary wall biosynthetic program. As a fiber crop, the cellulose is one of the main components of bast fiber. Cellulose synthase (CesA) is a key protein which regulates the cellulose biosynthetic process [24]. Identification of SSRs located in these TFs and CesAs is very useful in MAB. 97 polymorphic EST-SSRs, which were developed from TFs and CesAs’ sequences, could be regarded as potential functional markers for the improvement of jute fiber yield and quality.

## Supporting Information

S1 Fig Electrophoresis profile of CcES1743 marker for 12 jute accessions. M: 100 bp Ladder marker. 1: Huangma 179, 2: Aidianyehuangma, 3: Qiongyueqing, 4: D-154, 5: Yueyuan 5, 6: JRC-212, 7: BL/106CG, 8: Yunye1-1, 9: Kuanyechangguo, 10: Tianma, 11: Maliyeshengchangguo, 12: JRC/551.
(PPTX)

S1 Table Primers of 1,906 EST-SSR based on transcriptomic sequences in jute. (XLS)

S2 Table Gene annotation of 1,410unigenes contained SSR loci in jute. (XLS)

S3 Table Primers of 116 EST-SSR developed from TFs and CesAs’ sequences in jute. (XLS)

S4 Table Genetic similarity coefficient among 12 jute accessions by NTsysV2.02. (XLS)

## References

[1] Xiong HP. Breeding Sciences of Bast and Leaf Fiber Crops. 1st ed Beijing: Agricultral Science and Technology Press of China; 2008.

[2] Kundu A, Topdar N, Sarkar D, Sinha M, Ghosh A, Banerjee S, et al Origins of white (*Corchorus capsularis* L.) and dark (*C*. *olitorius* L.) jute: a reevaluation based on nuclear and chloroplast microsatellites. J Plant Biochem Biot. 2013; 22: 372–381.

[3] Mir R, Rustgi S, Sharma S, Singh R, Goyal A, Kumar J, et al A preliminary genetic analysis of fibre traits and the use of new genomic SSRs for genetic diversity in jute. Euphytica.2008; 161: 413–427.

[4] Chakraborty A, Sarkar D, Satya P, Karmakar P, Singh N. Pathways associated with lignin biosynthesis in lignomaniac jute fibres. Mol Genet Genomics. 2015; 290: 1523–1542. 10.1007/s00438-015-1013-y 25724692

[5] Biswas C, Dey P, Karmakar PG, Satpathy S. Discovery of large-scale SNP markers and construction of linkage map in a RIL population of jute (*Corchorus capsularis*). Mol Breeding. 2015; 35: 1–10.

[6] Das M, Banerjee S, Topdar N, Kundu A, Mir R, Sarkar D, et al QTL identification for molecular breeding of fibre yield and fibre quality traits in jute. Euphytica. 2012; 187: 175–189.

[7] Mir RR, Banerjee S, Das M, Gupta V, Tyagi AK, Sinha MK, et al Development and characterization of large-scale simple sequence repeats in jute. Crop Sci. 2009; 49: 1687–1694.

[8] Das M, Banerjee S, Dhariwal R, Vyas S, Mir R, Topdar N, et al Development of SSR markers and construction of a linkage map in jute. J Genet. 2012; 91: 21–31. 22546823 10.1007/s12041-012-0151-9

[9] Liu T, Zhu S, Fu L, Tang Q, Yu Y, Chen P, et al Development and characterization of 1,827 expressed sequence tag-derived simple sequence repeat markers for ramie (*Boehmeria niveaL*. *Gaud*). PLoS ONE. 2013; 8: e60346 10.1371/journal.pone.0060346 23565230 PMC3614921

[10] Gao C, Xin P, Cheng C, Tang Q, Chen P, Wang C, et al Diversity analysis in *Cannabis sativa*based on large-scale development of expressed sequence tag-derived simple sequence repeat markers. PLoS ONE. 2014; 9: e110638 10.1371/journal.pone.0110638 25329551 PMC4203809

[11] Saghai-Maroof MA, Soliman KM, Jorgensen RA, Allard RW. Ribosomal DNA spacer-length polymorphisms in barley: mendelian inheritance, chromosomal location, and population dynamics. P Natl Acad Sci USA. 1984; 81: 8014–8018. 10.1073/pnas.81.24.8014 PMC392284 6096873

[12] Rozen S, Skaletsky H. Primer3 on the WWW for general users and for biologist programmers. Methods Mol Biol. 2000; 132: 365–386. 10547847 10.1385/1-59259-192-2:365

[13] Zhang LW, Yuan MH, He XW, Liu X, Fang PP, Lin L, et al Development and universality evaluation of EST-SSR markers in jute (*Corchorus* spp.) from GenBank database. Acta Agron Sin.2014; 40: 1213–1219.

[14] Rohlf F. NTSYS-pc. Numerical taxonomy and multivariate analysis system, version 2.10. Exeter Software, New York. 2002.

[15] Liu K, Muse S. PowerMarker: an integrated analysis environment for genetic marker analysis. Bioinformatics.2005; 21: 2128–2129 15705655 10.1093/bioinformatics/bti282

[16] Cloutier S, Niu Z, Datla R, Duguid S. Development and analysis of EST-SSRs for flax (*Linum usitatissimum* L.). Theor Appl Genet. 2009; 119: 53–63. 10.1007/s00122-009-1016-3 19357828

[17] Aggarwal R, Hendre P, Varshney R, Bhat P, Krishnakumar V, Singh L. Identification, characterization and utilization of EST-derived genic microsatellite markers for genome analyses of coffee and related species. Theor Appl Genet. 2007; 114: 359–372. 17115127 10.1007/s00122-006-0440-x

[18] Peng JH, Lapitan NV. Characterization of EST-derived microsatellites in the wheat genome and development of eSSR markers. Funct Integr Genomic. 2005; 5: 80–96. 10.1007/s10142-004-0128-8 15650880

[19] Varshney RK, Grosse I, Hähnel U, Siefken R, Prasad M, Stein N, et al Genetic mapping and BAC assignment of EST-derived SSR markers shows non-uniform distribution of genes in the barley genome. Theor Appl Genet. 2006; 113: 239–250. 16791690 10.1007/s00122-006-0289-z

[20] Cheng X, Xu J, Xia S, Gu J, Yang Y, Fu J, et al Development and genetic mapping of microsatellite markers from genome survey sequences in *Brassica napus* . Theor Appl Genet. 2009;118: 1121–1131. 10.1007/s00122-009-0967-8 19190889

[21] Zhang T, Yuan Y, Yu J, Guo W, Kohel RJ. Molecular tagging of a major QTL for fiber strength in upland cotton and its marker-assisted selection. Theor Appl Genet. 2003; 106(2): 262–268. 12582851 10.1007/s00122-002-1101-3

[22] Andersen JR, Lübberstedt T. Functional markers in plants. Trends Plant Sci. 2003; 8: 554–560. 14607101 10.1016/j.tplants.2003.09.010

[23] Riechmann JL, Heard J, Martin G, Reuber L, Jiang C-Z, Keddie J, et al *Arabidopsis*transcription factors: genome-wide comparative analysis among eukaryotes. Science. 2000; 290: 2105–2110. 11118137 10.1126/science.290.5499.2105

[24] Doblin MS, Kurek I, Jacob-Wilk D, Delmer DP. Cellulose biosynthesis in plants: from genes to rosettes. Plant Cell Physiol. 2002; 43: 1407–1420. 12514238 10.1093/pcp/pcf164

