# Supplementary material for: Development and Characterization of 1,906 EST-SSR Markers from Unigenes in Jute (Corchorus spp.)
Source: PLoS One. 2015 Oct 29;10(10):e0140861. doi: 10.1371/journal.pone.0140861 (PMC4626149; doi:10.1371/journal.pone.0140861)
Supplement: S1 Fig — M: 100 bp Ladder marker. 1: Huangma 179, 2: Aidianyehuangma, 3: Qiongyueqing, 4: D-154, 5: Yueyuan 5, 6: JRC-212, 7: BL/106CG, 8: Yunye1-1, 9: Kuanyechangguo, 10: Tianma, 11: Maliyeshengchangguo, 12: JRC/551. (PPTX) [file pone.0140861.s001.pptx]

## Slide 1
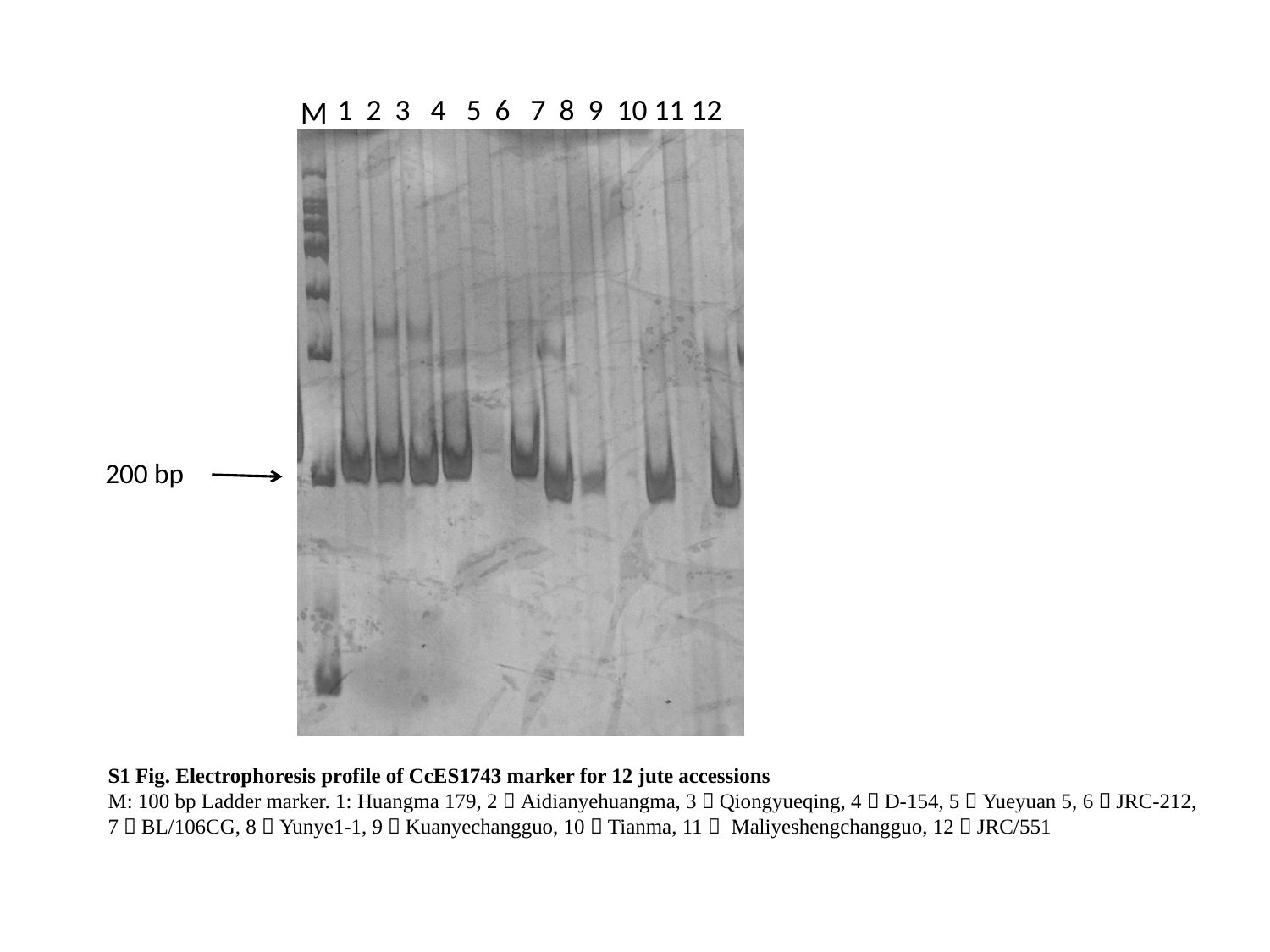

1 2 3 4 5 6 7 8 9 10 11 12
M
200 bp
S1 Fig. Electrophoresis profile of CcES1743 marker for 12 jute accessions
M: 100 bp Ladder marker. 1: Huangma 179, 2：Aidianyehuangma, 3：Qiongyueqing, 4：D-154, 5：Yueyuan 5, 6：JRC-212, 7：BL/106CG, 8：Yunye1-1, 9：Kuanyechangguo, 10：Tianma, 11： Maliyeshengchangguo, 12：JRC/551
